# Bioengineered Polyhydroxyalkanoates as Immobilized Enzyme Scaffolds for Industrial Applications

**DOI:** 10.3389/fbioe.2020.00156

**Published:** 2020-03-04

**Authors:** Jin Xiang Wong, Kampachiro Ogura, Shuxiong Chen, Bernd H. A. Rehm

**Affiliations:** ^1^School of Fundamental Sciences, Massey University, Palmerston North, New Zealand; ^2^MacDiarmid Institute of Advanced Materials and Nanotechnology, Victoria University of Wellington, Wellington, New Zealand; ^3^Centre for Cell Factories and Biopolymers, Griffith Institute for Drug Discovery, Griffith University, Nathan, QLD, Australia; ^4^Menzies Health Institute Queensland (MHIQ), Griffith University, Gold Coast Campus, Southport, QLD, Australia

**Keywords:** polyhydroxyalkanoate, enzyme immobilization, synthetic biology, industrial biotechnology, polymers

## Abstract

Enzymes function as biocatalysts and are extensively exploited in industrial applications. Immobilization of enzymes using support materials has been shown to improve enzyme properties, including stability and functionality in extreme conditions and recyclability in biocatalytic processing. This review focuses on the recent advances utilizing the design space of *in vivo* self-assembled polyhydroxyalkanoate (PHA) particles as biocatalyst immobilization scaffolds. Self-assembly of biologically active enzyme-coated PHA particles is a one-step *in vivo* production process, which avoids the costly and laborious *in vitro* chemical cross-linking of purified enzymes to separately produced support materials. The homogeneous orientation of enzymes densely coating PHA particles enhances the accessibility of catalytic sites, improving enzyme function. The PHA particle technology has been developed into a remarkable scaffolding platform for the design of cost-effective designer biocatalysts amenable toward robust industrial bioprocessing. In this review, the PHA particle technology will be compared to other biological supramolecular assembly-based technologies suitable for *in vivo* enzyme immobilization. Recent progress in the fabrication of biological particulate scaffolds using enzymes of industrial interest will be summarized. Additionally, we outline innovative approaches to overcome limitations of *in vivo* assembled PHA particles to enable fine-tuned immobilization of multiple enzymes to enhance performance in multi-step cascade reactions, such as those used in continuous flow bioprocessing.

## Enzyme Immobilization for Industrial Applications

Enzymes are capable of accelerating chemical reactions with high substrate specificity, stereoselectivity, and energy-efficient conversion properties (Robinson, 2015). These enzyme properties attract interest from the biotechnology sector and are considered as a substitute to chemical catalysts in various applications, such as biomass conversion, food processing, and the production of pharmaceuticals (Chapman et al., 2018). Despite the excellent catalytic properties of enzymes, utilization of natural enzymes at industrial scales is often hampered by their general protein characteristics (Mohamad et al., 2015). For example, enzymes are prone to denaturation/unfolding when removed from their native environments. In particular, enzymes are sensitive to changes in their environments and are poorly stable in extreme conditions, such as high temperatures, high pressures, extreme pHs, detergents, and organic solvents (Robinson, 2015). Furthermore, the complex reaction mixture makes it challenging to separate soluble enzymes and their respective products. Hence, enzymes are often rendered inactive and removed after a single use (Robinson, 2015). From an economic point of view, the poor reusability and difficulty in the recovery of enzymes are drawbacks, which severely limit the use of enzymes in industrial processes.

To overcome the shortcomings mentioned above, various enzyme immobilization techniques, especially scaffolding-based approaches, have been developed in the past decades (Cipolatti et al., 2017; Ren et al., 2019). Immobilization of enzymes results in the confinement of enzymes to a particular space, such as either displayed on, or encapsulated within, solid support materials, creating a heterogeneous biocatalyst system while retaining enzyme specificity and activity (Hartmeier, 2012). Interestingly, densely localizing enzymes on the scaffolding carriers can significantly improve enzymes’ catalytic performance and structural stability in certain scenarios due to macromolecular crowding (Yang et al., 2017; Zaak et al., 2017). The non-specific interactions between the immobilized enzymes and solid support materials could also further enhance the overall function and stability of immobilized enzymes (Fu et al., 2012; Rodrigues et al., 2013; Jia et al., 2017). The crowding of globular proteins could also create an artificial environment that can improve protein stability against chaotropic agents and temperature stress (Minton, 2000).

Immobilized enzyme-based catalytic systems facilitate separation of the enzyme from the reaction mixture. This strategy enables the repeated use of the immobilized enzymes and rapid termination of a catalytic reaction by physically removing the immobilized enzyme-bearing carriers from the reaction mixture (Mohamad et al., 2015; Bernal et al., 2018). This approach also prevents contamination of the product by the carried-over enzyme, thus reducing downstream process complexity and operational costs. Moreover, immobilized enzyme-based biocatalysts allow the implementation of flow-through formats in continuous bioprocessing approaches (Homaei et al., 2013; Zdarta et al., 2018). Nevertheless, in some cases, enzyme immobilization can impair the functionality of enzymes, as a result of unfavorable conformational changes in enzymes and restricted substrate access in comparison to their soluble counterpart (Guisan, 2006; Cao et al., 2012; Fernandez-Lopez et al., 2017). However, the advantages of enzyme immobilization outweigh their unfavorable impact and enhance the efficient implementation of biocatalysts in industrial processes.

Therefore, it is paramount to develop cost-effective and pragmatic enzyme immobilization approaches for potential industrial applications (Rehm et al., 2016, 2018; Nguyen and Kim, 2017). In general, scaffolding-based enzyme immobilization strategies can be categorized into *in vitro* and *in vivo* approaches. The *in vitro* approaches can offer excellent controllability by tuning the physicochemical properties of carriers (e.g., particle size and distribution, or surface charge) as well as by controlling the density of the immobilized enzymes (Faccio, 2018; Gonzalez-Miro et al., 2019). However, the *in vitro* methods often require harsh reaction conditions, such as the presence of toxic cross-linking agents, solvents, extreme temperatures, and pHs, for successful enzyme immobilization, and these conditions can potentially compromise enzyme function (Sletten and Bertozzi, 2009). Furthermore, most *in vitro* immobilization methods (e.g., chemical modifications and physical adsorption) are not able to control the enzyme orientation on the solid supports, which directly influences the accessibility of substrates to the catalytic sites of enzymes (Hess et al., 2012; Brune et al., 2016). Also, due to the inherent structural complexity of the enzymes, localizing them onto support materials using existing *in vitro* conjugation technologies often necessitates labor-intensive reactions and process optimization steps (Krauss et al., 2017; Rehm et al., 2018). In addition, multiple separate manufacturing schemes are necessary for large-scale manufacturing of biocatalysts using *in vitro* cross-linking technologies (e.g., manufacturing lines for both enzyme and support materials, and subsequent conjugation steps), which increases production cost (Krauss et al., 2017; Rehm et al., 2018; Zdarta et al., 2018).

Recently developed *in vivo* immobilization strategies offer an exciting new concept for enzyme immobilization that holds the promise for cost-effective production of improved industrial biocatalysts (Rehm et al., 2018). Recent progress in understanding the underlying self-assembly mechanism of a diverse range of naturally occurring supramolecular nanostructures has led to the possibility of constructing task-specific designer scaffolding platforms *in vivo*. Industrially relevant enzymes of interest can be covalently displayed on the surface and/or incorporated easily within a variety of bio-nanostructures *in vivo* by genetic engineering of the self-assembling subunits (Rehm et al., 2016; Wilkerson et al., 2018; Schmid-Dannert and López-Gallego, 2019). In contrast to the *in vitro* methods, the *in vivo* approaches can display enzymes in a homogeneous and oriented manner on solid supports. These *in vivo* approaches enable to bypass the harsh and time-consuming immobilization procedures that are often encountered in the *in vitro* methods. The *in vivo* formation of solid supports displaying enzymes is implemented intracellularly in bacterial cells by one-step production and, thus, additional cross-linking between the enzymes and solid materials is not needed. This one-pot approach is convenient, efficient, and ultimately enables the low-cost production of robust biocatalysts at a large scale (Rehm et al., 2016).

**FIGURE 1:**
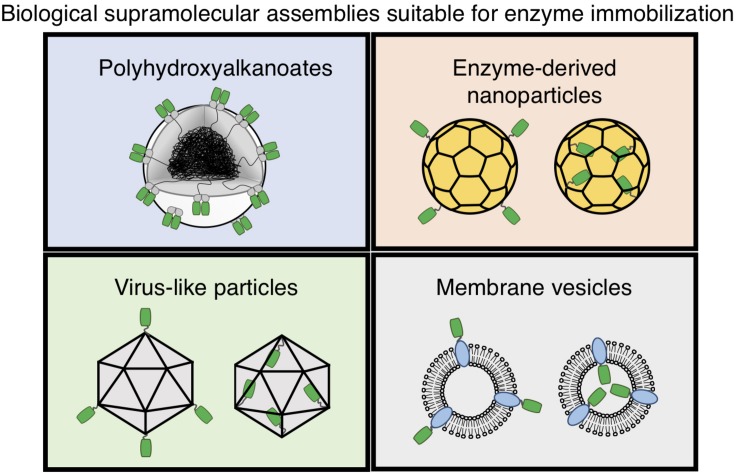
Enzyme (shown in green) immobilization via various biological supramolecular assemblies.

Several promising biological supramolecular assemblies, such as polyhydroxyalkanoate (PHA) particles (Hooks et al., 2014; Parlane et al., 2016b), virus-like particles (VLPs) (Schwarz et al., 2017; Wilkerson et al., 2018), enzyme-derived nanoparticles (EZPs) (Raeeszadeh-Sarmazdeh et al., 2016; Diaz et al., 2018; Schmid-Dannert and López-Gallego, 2019), membrane vesicles (Rehm et al., 2016; Sharma et al., 2018), and magnetosomes (Jacob and Suthindhiran, 2016; Yan et al., 2017) have been studied to immobilize a variety of functional proteins, including industrially relevant enzymes using recombinant fusion technology (Figure 1). Briefly, genetically amenable components of these scaffolds are translationally fused with proteins of interest, such as, e.g., enzymes, and are produced in a range of recombinant expression systems, like various prokaryotic and eukaryotic organisms. These recombinant host cells allow simultaneous protein and scaffold synthesis and subsequent self-assembly of these components. These methods have shown their applicability in the production of immobilized enzymes with improved functionality, presenting a promising means for cost-effective and one-step *in vivo* enzyme immobilization. Here, we will first review the most promising supramolecular assemblies suitable for *in vivo* enzyme immobilization and their recent proof-of-concept demonstrations. Then, we will compare the advantages and limitations of PHA particle technology with other biological scaffold-based *in vivo* enzyme immobilization methods focusing on immobilization of industrially relevant enzymes. Finally, we will discuss innovative methods to expand the utility of the PHA particle technology, including its implementation into continuous-flow catalytic conversions.

## Utilization of Various Supramolecular Assemblies as Enzyme Immobilization Supports

### Polyhydroxyalkanoate

Polyhydroxyalkanoates are natural biopolyesters, composed of (*R*)-3-hydroxy fatty acids, and are produced by various bacteria in the presence of an excess carbon source, such as glucose (Gonzalez-Miro et al., 2019; Moradali and Rehm, 2020). PHAs are synthesized by PHA synthases and are deposited as spherical polyester inclusions, which serve as an energy and carbon source (Rehm, 2003, 2010). PHA particles vary in size and range between 100 and 500 nm (Steinmann et al., 2010; Parlane et al., 2016b; Gonzalez-Miro et al., 2019). Poly-(*R*)-3-hydroxybutyrate (PHB) was the first PHA polymer identified by Lemoigne in 1926 in *Bacillus megaterium* and is the most common form of PHA (Kikkawa et al., 2005; Campisano et al., 2008). Generally, each bacterial cell can produce 5–10 PHA particles, the mass of which can contribute up to 90% of cellular dry weight (Lee, 1996; Koller et al., 2010; Mathuriya and Yakhmi, 2017). The physicochemical properties of PHA particles are significantly influenced by the length and composition of the hydroxyl fatty acids (Mathuriya and Yakhmi, 2017; Gonzalez-Miro et al., 2019). Over 150 different PHA constituents are known (Rehm, 2007; Keshavarz and Roy, 2010; Gonzalez-Miro et al., 2019). The PHAs are classified into three main classes, dependent on the chemical structure and the chain length of the fatty acid monomers: short-chain length PHAs (three to five carbon atoms); medium-chain length PHAs (6–14 carbon atoms); and long-chain length PHAs (>14 carbon atoms) (Mathuriya and Yakhmi, 2017; Ross et al., 2017). Short-chain length PHAs generally have a high level of crystallinity and, thus, are hard and brittle. Medium-chain length PHAs usually have a low melting temperature and crystallinity and, therefore, they are more elastomeric (Philip et al., 2007; Parlane et al., 2016b; Mathuriya and Yakhmi, 2017).

**FIGURE 2:**
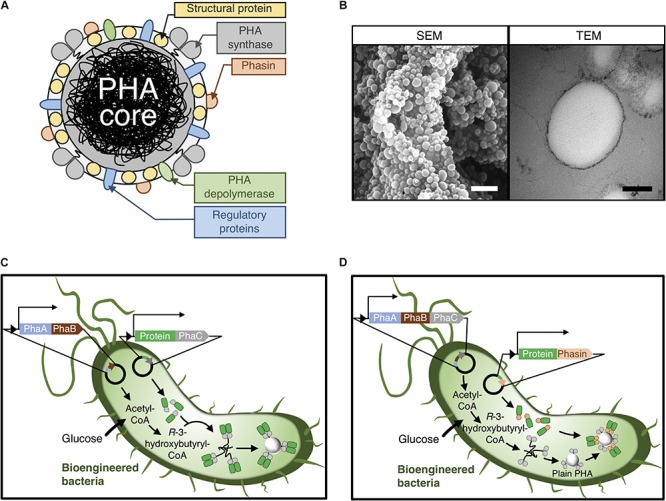
**(A)** Schematic of a wild-type PHA particle coated by PAPs. **(B)** SEM and TEM micrographs of PHA particles. White scale bar, 1 μm; black scale bar, 100 nm. **(C)** Self-assembly and functionalization of PHA particles using a PhaC-based gene fusion approach. **(D)** Self-assembly and functionalization of PHA particles using a phasin-based translational fusion approach.

Polyhydroxyalkanoate particles are comprised of an amorphous hydrophobic PHA core surrounded by PHA-associated proteins (PAPs), including PHA synthase (PhaC), phasins (e.g., PhaP and PhaF), structural proteins, PHA depolymerase, structural proteins, and other regulatory proteins (Figure 2A) (Parlane et al., 2016b). Numerous metabolic pathways can provide an array of (*R*)-3-hydroxy fatty acids for the production of PHAs with varying structures and properties as reviewed elsewhere (Meng et al., 2014). PhaC dimers can polymerize these monomer precursors to PHA chains while PhaC itself remains attached to nascent PHA chains via a covalent thioester bond involving the active site cysteine residue of the PhaC (Peoples and Sinskey, 1989a, b). The covalent link between these two components, namely, the growing hydrophobic PHA chains and the soluble PhaC, eventually leads to amphipathic molecules self-assembling into the spherical PHA particles as shown in scanning electron microscopy (SEM) and transmission electron microscopy (TEM) micrographs (Figure 2B) (Taguchi and Doi, 2004; Rehm, 2007). Another interesting class of PAPs, the phasins, are a type of amphipathic proteins that have several roles in controlling the structure and surface properties of PHA particles (Pieper-Fürst et al., 1994; Wieczorek et al., 1995). Notably, phasins have a high binding affinity to the outer surface of PHA particles *in vivo* and *in vitro* mediated by physical adsorption (Tarazona et al., 2019).

The PHA-anchoring characteristics of these PAPs via both covalent interactions (PhaC) and physical adsorption (PhaF and PhaP) to the surface of PHA particles have been exploited to fabricate task-specific designer PHA particles using recombinant DNA technology (Hay et al., 2015; Hooks and Rehm, 2015; Jahns and Rehm, 2015; Bello-Gil et al., 2018a, b). PAPs can be translationally fused to target proteins, including industrially relevant enzymes, to enable the recombinant production of functionalized PHA particles *in vivo* (Figures 2C,D). This approach allows the cost-effective oriented display of immobilized enzymes on the polymeric particulate carrier in one step, ultimately avoiding the laborious chemical cross-linking between enzymes and particles *in vitro* after isolation (Grage et al., 2009; Hooks et al., 2014; Parlane et al., 2016b).

### Virus-Like Particles (VLPs)

Virus-like particles consist of the viral capsid proteins (CPs; Charlton Hume et al., 2019). The formation of VLPs is a self-assembling process of the viral capsid, which potentially mimics the general structure of the parental virus. However, VLPs do not contain nucleic acids, and, thus, there is no risk of causing infection (Charlton Hume et al., 2019; Janitzek et al., 2019). The CP subunits can be genetically modified for bioconjugation, enabling molecules of interest to be densely displayed or encapsulated in homogeneous spatial orientation (Tan and Jiang, 2017; Charlton Hume et al., 2019). VLPs have made significant advances in various fields, from vaccinology to industrial uses due to their promising characteristics, including monodispersed particle size distribution, defined geometric surfaces, biosafety, and functional programmability (Mohsen et al., 2017; Janitzek et al., 2019). Additionally, the viral capsids are stable over a wide range of environmental conditions, such as temperatures and pHs, which make them suitable for different applications, including industrial biocatalysis (Stanley, 2014; Koch et al., 2015; Brasch et al., 2017; Torbensen et al., 2019). Nevertheless, VLPs are challenging to be abundantly produced at the industrial scale (Mohsen et al., 2017; Tan and Jiang, 2017). A significant drawback of the VLP platform is that the size of the protein attached to, or accommodated within, the particles is limited. This disadvantage precludes the presentation of large functional moieties (Charlton Hume et al., 2019).

### Enzyme-Derived Nanoparticles (EZPs)

Enzyme-derived nanoparticles are highly organized cage-like nanostructures that can be found in both prokaryotic and eukaryotic cells. These naturally evolved protein assemblies can often comprise biomacromolecules such as, e.g., enzymes or inorganic moieties (e.g., iron) that are involved in a range of metabolic and biochemical pathways [peroxidase catalyzed processes (encapsulin), production of vitamin B2 (lumazine synthase), and iron homeostasis (ferritin)] (Arosio et al., 2009; Giessen and Silver, 2017; Azuma et al., 2018). These spherical nanostructures are highly attractive owing to their particle uniformity, biocompatibility, and precise controllability. Being able to fine-tune the morphological architecture and functions of these particulate scaffolds has made them excellent candidates for the design of biocatalytic nanoreactors (Raeeszadeh-Sarmazdeh et al., 2016; Lee, 2018; Schmid-Dannert and López-Gallego, 2019). EZPs can be reprogrammed to incorporate various foreign biological functions such as, e.g., enzymes of industrial interest. Both chemical and bioengineering methods can be utilized to modify the scaffold protein (SP) subunits of EZPs to enable spatial organization of enzymes within and/or on the surface of the EZPs. This design space enables the fabrication of various artificial multienzyme complexes for industrial uses (Schmid-Dannert and López-Gallego, 2019). Although these scaffolds have been manufactured in numerous recombinant expression systems (e.g., various prokaryotic and eukaryotic organisms), they have been mainly assembled in *Escherichia coli* strains (Diaz et al., 2018). Advances in protein engineering in recent years harnessed the unique structural assembly of EZPs to enable the *de novo* and *in silico* design of novel EZPs (King et al., 2014; Bale et al., 2016; Hsia et al., 2016; Jakobson et al., 2017; Xu et al., 2019).

### Extracellular Membrane Vesicles (EMVs)

Extracellular membrane vesicles are lipid membrane-derived compartments and are found in all domains of life (Raposo and Stoorvogel, 2013; Gill et al., 2018; Lee, 2019). Their sizes are in the range of 20–1000 nm in diameter (van der Pol et al., 2012; Gill et al., 2018), and they mainly serve as carrier vehicles to mediate cell-to-cell communication by transporting biological cargo as, for example, DNA, RNA, and proteins (Burkova et al., 2018; Longatti et al., 2018). The classification of these functionally and structurally diverse EMVs, including the bacterial outer membrane vesicles, microvesicles, and exosomes, has been thoroughly reviewed (van der Pol et al., 2012; Cocucci and Meldolesi, 2015; Gill et al., 2018; Greening and Simpson, 2018). Although the exact underlying mechanism on how different EMVs are formed is still unknown, recent studies show that various recombinantly modified protein production cell lines, including well-established *E. coli* production strains, can produce task-specific EMVs. It was shown that foreign proteins of interest, such as enzymes, could be incorporated into the outer surface or within the inner surface of the EMVs via membrane-anchoring motifs, such as transmembrane domains, using genetic engineering to create respective translational fusions (Alves et al., 2015; Su et al., 2017). This approach led to numerous pharmaceutical and bioremediation applications (Ohno et al., 2013; Alves et al., 2016, 2018; Arrighetti et al., 2019). In addition, EMVs are relatively stable in ambient environments and can be manufactured cost-effectively (Collins, 2011). However, isolation and purification of EMVs still require expensive and laborious ultracentrifugation steps, which potentially impact the structural integrity of EMVs and which prohibit industrial scale production (Su et al., 2017; Longatti et al., 2018; Bruce et al., 2019).

### Magnetosomes

Bacterial magnetosomes are inclusions (20–60 nm) present in magnetotactic bacteria comprised of magnetic mineral crystals (iron oxide or iron sulfide nanoparticle) surrounded by a phospholipid double-layered membrane (Dieudonné et al., 2019). The magnetosome membrane is derived from the cytoplasmic membrane and can protect the iron crystal from oxidation (Dieudonné et al., 2019). Many magnetosome membrane proteins (e.g., MamB, MamM, MamH, and MamZ) are involved in magnetosome formation and dictate the iron uptake into the vesicle (Lohße et al., 2014; Uebe and Schüler, 2016; Dieudonné et al., 2019). Meanwhile, the size and morphology of the magnetosomes are controlled by another set of magnetosome membrane proteins as, for example, MamC/Mms13, MamD, MamF, MamG, MamR, MamS, Mms6, and MmsF (Uebe and Schüler, 2016; Islam et al., 2018; Dieudonné et al., 2019). Interestingly, magnetosomes can be functionalized *in vivo* by fusing foreign proteins of interest to the magnetosome membrane proteins, such as MamC/Mms13, MagA, and Mms16 (Arakaki et al., 2008; Ren et al., 2018). The translational fusion of functional proteins to these transmembrane proteins of magnetosomes has led to numerous successful prototypes in a wide range of applications, including of industrial uses (Ginet et al., 2011; Sugamata et al., 2013; Honda et al., 2015a, b; Xiang et al., 2017; Mickoleit and Schüler, 2018). The inherent magnetic characteristics of magnetosomes make them very useful in some situations, especially for implementation in magnetic-field-related technologies, such as magneto-immunoassays and biomedical imaging (Ren et al., 2018). The implementation of magnetosomes also allows rapid magnetic separation of the functionalized particulate scaffolds from the bulk fluids (Rehm et al., 2018). However, several technical issues encountered in manipulating and cultivating magnetosomes represent some of the main hurdles in expanding the use of this exciting technology (Bakhshi et al., 2016).

## Biological Supramolecular Assemblies as Biocatalyst Supports

Table 1 summarizes recent studies describing *in vivo* immobilization approaches for a range of industrially relevant enzymes, detailing their functional performance and robustness in various experimented conditions.

**TABLE 1:** Biological supramolecular assemblies engineered for *in vivo* immobilization of industrially relevant enzymes.

**Type of biological scaffolds and their anchoring motifs**	**Target enzyme (Origin) (Gene fusion site) (Production host)**	**Catalytic performance**	**Stability**	**References**
**Polyhydroxyalkanoates (PHAs)**				
PHAs via *Cupriavidus necator* PHA synthase PhaC	α-Amylase (*Bacillus licheniformis*) (C-terminus) [*E. coli* Origami B (DE3)]	• Consistent with the reported activity of soluble counterpart. • Michaelis–Menten constant (*K*_*m*_) of immobilized α-amylase catalyzing starch degradation: 5 μM. • *K*_*m*_ of soluble α-amylase reported in the literature catalyzing starch degradation: 9.6 μM. • Specific activity of immobilized α-amylase catalyzing starch degradation: 506 mU/mg of fusion protein.	• Tolerant to extreme pH and temperature conditions.	Rasiah and Rehm, 2009
	Hexavalent chromium reductase, NemA (*E. coli*) (N-terminus) [*E. coli* BL21(DE3)]	• Showed activity to their substrate but at varying efficiencies. *K*_*m*_ of immobilized NemA for the reduction of Cr(VI): 94 ± 26 μM. • *K*_*m*_ of soluble NemA for the reduction of Cr(VI): 16 ± 8.6 μM. • *K*_*m*_ of immobilized NemA for the reduction of NADH: 490 ± 30 μM. • *K*_*m*_ of soluble NemA for the reduction of NADH: 450 ± 30 μM	• No observable reduction in activity after 36 weeks of storage at 4°C.	Robins et al., 2013^∧^
	*N*-acetylglucosamine 2-epimerase, Slr1975 (*Synechocystis* sp. PCC 6803) (N-terminus) [*E. coli* BL21(DE3)]	• Artificial enzyme cascading system had overall conversion yield of ∼22%, compared to that of traditional method at ∼33% catalyzing *N*-acetyl-D-glucosamine conversion to *N*-acetylneuraminic acid. • Specific activity of immobilized Slr1975 catalyzing *N*-acetyl-D-glucosamine conversion to *N*-acetyl-D-mannosamine: 1.76 ± 0.38 U/mg fusion protein. • Specific activity of immobilized Slr1975 catalyzing *N*-acetyl-D-glucosamine conversion to *N*-acetyl-D-mannosamine when co-immobilized with NanA: 0.58 ± 0.07 U/mg of fusion protein. • Specific activity of immobilized NanA catalyzing *N*-acetyl-D-mannosamine conversion to *N*-acetylneuraminic acid: 42.6 ± 6.9 U/mg of fusion protein. • Specific activity of immobilized NanA catalyzing *N*-acetyl-D-mannosamine conversion to *N*-acetylneuraminic acid when co-immobilized with Slr1975: 81.9 ± 19 U/mg of fusion protein.	• Retained ∼80% of its initial activity after five reaction cycles.	Hooks et al., 2013*
	*N*-acetylneuraminic acid aldolase, NanA (*E. coli*) (C-terminus) [*E. coli* BL21(DE3)]			
	Lipase B (*Candida antarctica*) (N-terminus) [*E. coli* BL21 (DE3)]	• Retained but exhibited lower activity (∼30–40%) catalyzing glycerol tributyrate hydrolysis when compared to the commercially available immobilized lipase (Novozyme 435).	• Retained initial activity after 7 weeks of storage at 4°C.	Jahns and Rehm, 2015^∧^*
	Carbonic anhydrase (*Desulfovibrio vulgaris* str. “Miyazaki F”), DvCA (C-terminus) [*E. coli* BL21(DE3)]	• Retained but exhibited lower activity when compared to the commercially available soluble counterpart. • Specific activity of immobilized DvCA catalyzing the hydration of carbon dioxide: 114 U/mg of enzyme (highest at 211 U/mg of enzyme).	• Tolerant to alkaline and elevated temperature environments.	Hooks and Rehm, 2015*
	Lipase M37 (*Photobacterium lipolyticum*) (C-terminus) (*E. coli* XL1-Blue)	• Consistent with the reported activity of soluble counterpart but exhibited narrow substrate chain length specificity. • Specific activity of immobilized lipase M37 catalyzing *p*-nitrophenyl esters conversion to *p*-nitrophenol: 108.4 ± 2.5 U/g of dry weight PHA particles.	• Enhanced thermal stability and retained initial activity after 4 weeks of storage at 4°C.	Yang et al., 2015*
	Alkaline polygalacturonate lyase, PGL (*Bacillus subtilis*) (C-terminus) [*E. coli* BL21(DE3)]	• Retained ∼85% of the catalytic activity of soluble counterpart. • Specific activity of immobilized PGL catalyzing polygalacturonic acid conversion to unsaturated oligo-galacturonic acid: 184.67 ± 11.53 U/mg of enzyme. • Specific activity of soluble PGL catalyzing polygalacturonic acid conversion to unsaturated oligo-galacturonic acid: 215.93 ± 8.95 U/mg of enzyme.	• Retained ∼60% of its initial activity after eight reaction cycles. • Moderately enhanced thermal and pH stability.	Ran et al., 2017*
	Tyrosinase (*Verrucomicrobium spinosum*) (C-terminus) [*E. coli* BL21(DE3)]	• Immobilized tyrosinase showed enhanced specific activity catalyzing L-tyrosine conversion to L-dopaquinone when compared to its soluble counterpart. • Monophenolase activity of immobilized tyrosinase catalyzing L-tyrosine conversion to 3,4-dihydroxyphenyl-L-alanine: 9155.88 ± 312.57 U/g of enzyme. • Monophenolase activity of soluble tyrosinase catalyzing L-tyrosine conversion to 3,4-dihydroxyphenyl-L-alanine: 2185.50 ± 74.61 U/g of enzyme. • Diphenolase activity of immobilized tyrosinase catalyzing 3,4-dihydroxyphenyl-L-alanine conversion to L-dopaquinone: 297.27 ± 21.25 U/g of enzyme. • Diphenolase activity of soluble tyrosinase catalyzing 3,4-dihydroxyphenyl-L-alanine conversion to L-dopaquinone: 32.10 ± 3.10 U/g of enzyme.	• Retained its initial activity up to six reaction cycles. • Widened optimal operating temperature range.	Tan et al., 2019*
	D-tagatose-3-epimerase, DTE (*Pseudomonas cichorii*) (C-terminus) [*E. coli ClearColi* BL21 (DE3)]	• Had overall conversion yield of ∼33% catalyzing D-fructose conversion to D-allulose. • Specific activity of immobilized DTE catalyzing D-fructose conversion to D-allulose: 357.77 ± 16.66 U/mg of enzyme. • Specific activity of soluble DTE catalyzing D-fructose conversion to D-allulose: 531.29 ± 31.87 U/mg of enzyme.	• Retained ∼80% of its initial activity after eight reaction cycles. • Exhibited similar thermal and pH stability when compared to its soluble counterpart.	Ran et al., 2019*
PHAs via *Pseudomonas putida* phasin PhaF	β-Galactosidase, β-gal (*E. coli*) (N-terminus) (*Pseudomonas putida* GPG-Tc6)	• Showed specific activity to its substrate. • Specific activity of immobilized β-gal catalyzing the hydrolysis of *o*-nitro-phenyl-β-D-galactopyranoside: 2.8 × 10^5^ U/mg of enzyme. • Specific activity of soluble β-gal catalyzing the hydrolysis of *o*-nitro-phenyl-β-D-galactopyranoside cleaved from β-gal displaying PHA particles: 2.2 × 10^5^ U/mg of enzyme.	• N/A	Moldes et al., 2004*
	Cry1Ab toxin (*Bacillus thuringiensis*) (N-terminus) (*Pseudomonas putida* GPG-Tc6)	• Immobilized Cry1Ab showed 7.2-fold less insecticidal activity against the larvae of *Sesamia nonagrioides* when compared with its soluble counterpart.	• N/A	Moldes et al., 2006^∧^*
PHAs via *Cupriavidus necator* phasin PhaP	D-hydantoinase, D-HDT (*Agrobacterium radiobacter* NRRL B11291) (N-terminus) (*E. coli* DH5α)	• Immobilized D-HDT showed similar specific activity in catalyzing D,L-hydroxyphenyl hydantoin conversion to *N*-carbamoyl-L-*p*-hydroxy phenylglycine with its soluble counterpart. • Ranged between 80 and 107 U due to varying biosynthesis conditions of *in vivo* functionalized PHA particles.	• Stable up to seven reaction cycles. Enhanced stability at elevated temperatures.	Chen S. Y. et al., 2014*
	Lysine decarboxylase, CadA (*E. coli*) (N-terminus) [*E. coli* BL21(DE3)]	• Consistent with its soluble counterpart. • Specific activity of immobilized CadA catalyzing lysine conversion to cadaverine: 179.5 ± 1.8 U/mg of enzyme. • Specific activity of soluble CadA catalyzing lysine conversion to cadaverine: 95.15 ± 9.5 U/mg of enzyme.	• Retained its initial activity up to five reaction cycles. • Moderately enhanced thermal and pH stability.	Seo et al., 2016*
PHAs via *Cupriavidus necator* PHA synthase PhaC and PHAs via *Cupriavidus necator* phasin PhaP	Organophosphorus hydrolase, OpdA (*Pseudoalteromonas* sp. SCSIO 04301) (N-terminus) [*E. coli* BL21(DE3)]	• *K*_*m*_ of OpdA immobilized using PhaC catalyzing paraoxon hydrolysis: 6.188 ± 2.490 mM. • *K*_*m*_ of OpdA immobilized using PhaP catalyzing paraoxon hydrolysis: 6.116 ± 1.299 mM. • *K*_*m*_ of soluble OpdA catalyzing paraoxon hydrolysis: 3.203 ± 0.929 mM. • *k*_*cat*_ of OpdA immobilized using PhaC catalyzing paraoxon hydrolysis: 11.904 ± 3.893 s^–1^. • *k*_*cat*_ of OpdA immobilized using PhaP catalyzing paraoxon hydrolysis: 11.223 ± 1.752 s^–1^. • *k*_*cat*_ of soluble OpdA catalyzing paraoxon hydrolysis: 3.0 ± 0.526 s^–1^. • *k_*cat*_/K_*m*_* of OpdA immobilized using PhaC catalyzing paraoxon hydrolysis: 1961 ± 138 M^–1^s^–1^. • *k_*cat*_/K_*m*_* of OpdA immobilized using PhaP catalyzing paraoxon hydrolysis: 1850 ± 104 M^–1^s^–1^. • *k_*cat*_/K_*m*_* of soluble OpdA catalyzing paraoxon hydrolysis: 935 ± 89 M^–1^s^–1^. • Specific activity of OpdA immobilized using PhaC catalyzing paraoxon hydrolysis: 0.096 ± 0.0047 U/mg of enzyme. • Specific activity of OpdA immobilized using PhaP catalyzing paraoxon hydrolysis: 0.109 ± 0.0014 U/mg of enzyme. • Specific activity of OpdA immobilized using PhaC and PhaP catalyzing paraoxon hydrolysis: 0.112 ± 0.0044 U/mg of enzyme. • Specific activity of soluble OpdA catalyzing paraoxon hydrolysis: 1.648 ± 0.222 U/mg of enzyme.	• Enhanced stability particularly under acidic conditions. • Retained ∼80% of its initial activity after 10 repeated use cycles.	Li et al., 2019
**Virus-like particles (VLPs)**				
Bacteriophage MS2 CP subunit	Pyridoxal phosphate-dependent tryptophanase, TnaA (*E. coli*) (N- and C-termini) [*E. coli* BL21(DE3) Star]	• Artificial enzyme cascading system comprised of covalently immobilized TnaA and FMO showed enhanced overall conversion yield catalyzing L-tryptophan conversion to indigo when compared to the soluble controls.	• Retained ∼95% of its initial activity after 1 week of storage at 25°C, compared to its soluble counterpart (∼5%).	Giessen and Silver, 2016^∧^*
	Flavin-mononucleotide and nicotinamide adenine dinucleotide phosphate dependent containing monooxygenase, FMO (*Methylophaga* sp. Strain SK1) (N- and C-termini) [*E. coli* BL21(DE3) Star]			
Bacteriophage P22 CP subunit	Alcohol dehydrogenase D (*Pyrococcus furiosus*) (C-terminus) [*E. coli* BL21(DE3)]	• Showed specific activity for the reduction of 3-hydroxy-2-butanone to 2,3-butanediol.	• No loss in activity at 25°C was observed.	Patterson et al., 2015^∧^*
	Hydrogenase 1 subunit A and subunit B, HyaA and HyaB (*E. coli*) (C-terminus) [*E. coli* BL21(DE3)]	• ∼80–270-fold higher than the reported activity of soluble counterpart for hydrogen production. • Catalytic activity of immobilized hydrogenase for hydrogen production: 3218 ± 394 nmol H_2_/mg min. • Catalytic activity of the soluble hydrogenase for hydrogen production reported in the literature: 12–38 nmol H_2_/mg min.	• Showed resistance against proteolytic and thermal inactivation.	Jordan et al., 2016*
Parvovirus B19 CP subunit	Lipase, Bp1A (*Bacillus pumilus*) (N- and C-termini) [*E. coli* BL21(DE3)]	• Showed specific activity catalyzing the hydrolysis of 4-nitrophenyl acetate but lower when compared to its soluble counterpart. • Specific activity of immobilized Bp1A catalyzing the hydrolysis of 4-nitrophenyl acetate: 9.5 ± 1.4 U/μmol of enzyme. • Specific activity of soluble Bp1A catalyzing the hydrolysis of 4-nitrophenyl acetate: 202 ± 0.4 U/μmol enzyme.	• Enhanced thermal stability. • First-order rate constant of degradation of immobilized lipase at 40°C: 0.68 ± 0.11 h^–1^. • First-order rate constant of degradation of soluble lipase at 40°C: 4.82 ± 0.37 h^–1^.	Bustos-Jaimes et al., 2017*
	α-Glucosidase, Ima1p (*Saccharomyces cerevisiae*) (C-terminus) [*E. coli* BL21(DE3)]	• ∼Threefold increase in catalytic activity when compared to its soluble counterpart. • Catalytic activity of immobilized Ima1p catalyzing 4-nitrophenyl-α-D-glucopyranoside hydrolysis: 2.1 ± 0.05 mM/min/mg. • Catalytic activity of soluble Ima1p catalyzing 4-nitrophenyl-α-D-glucopyranoside hydrolysis: 0.67 ± 0.02 mM/min/mg. • *K*_*m*_ of immobilized Ima1p catalyzing 4-nitrophenyl-α-D-glucopyranoside hydrolysis: 1.92 ± 0.13 mM. • *K*_*m*_ of soluble Ima1p catalyzing 4-nitrophenyl-α-D-glucopyranoside hydrolysis: 1.72 ± 0.16 mM.	• Impaired thermal stability.	Cayetano-Cruz et al., 2018
Cowpea chlorotic mottle virus CP subunit	Lysozyme (Enterobacteria phage T4) (C-terminus) [*E. coli* BLR(DE3) pLysS]	• Showed catalytic activity catalyzing the degradation of fluorescently labeled *M. luteus* cell walls but ∼7-fold less active than its soluble counterpart. • Catalytic activity of immobilized lysozyme catalyzing the degradation of fluorescently labeled *M. luteus* cell walls: ∼400 arbitrary unit (AU)/min. • Catalytic activity of soluble lysozyme catalyzing the degradation of fluorescently labeled *M. luteus* cell walls: ∼2800 AU/min.	• N/A	Schoonen et al., 2017*
**Enzyme-derived nanoparticles (EZPs)**				
*Bacillus stearothermophilus* pyruvate dehydrogenase multienzyme complex E2 core SP subunit functionalized with elastin-like peptide (ELP-E2)	Endoglucanase CelA (*Clostridium thermocellum*) (C-terminus) [*E. coli* BL21(DE3)]	• Immobilized CelA on ELP-E2 nanoparticles increased the amount of reduced sugar compared to its soluble counterpart. • Catalytic activity of immobilized CelA catalyzing cellulose hydrolysis: ∼17 μmol/h. • Catalytic activity of soluble CelA catalyzing cellulose hydrolysis: ∼14 μmol/h.	• Immobilized CelA on ELP-E2 nanoparticles remained functional up to 70°C.	Chen et al., 2015*
	β-Galactosidase, β-gal (*E. coli*) (C-terminus) [*E. coli* BL21(DE3)]	• Immobilized β-gal on ELP-E2 nanoparticles showed catalytic activity visualized by the change in the color of substrate into yellow due to the release of *o*-nitrophenol.	• N/A	
*Citrobacter freundii* Pdu bacterial microcompartment SP subunit (D18 or P18)	Glycerol dehydrogenase, GldA (*E. coli*) (N-terminus) [*E. coli* BL21(DE3) pLysS] Dihydroxyacetone kinase, DhaK (*E. coli*) (N-terminus) [*E. coli* BL21(DE3) pLysS] Methylglyoxal synthase, MgsA (*E. coli*) (N-terminus) [*E. coli* BL21(DE3) pLysS] 1,2-propanediol oxidoreductase, FucO (*E. coli*) (N-terminus) [*E. coli* BL21(DE3) pLysS]	• Co-immobilization or aggregation of tagged enzymes catalyzing glycerol conversion to 1,2-propanediol resulted in enhanced conversion yield *in vivo* compared to the soluble counterpart. • A reduction of 90% in the specific activity of GldA bearing D18 when compared to the untagged control catalyzing glycerol conversion to dihydroacetone. • A reduction of 55% in the specific activity of GldA bearing P18 when compared to the untagged control catalyzing glycerol conversion to dihydroacetone. • Specific activity of immobilized DhaK bearing D18 catalyzing dihydroacetone conversion to dihydroacetone phosphate: ∼5.5 μmol/min/mg. • Specific activity of immobilized DhaK bearing P18 catalyzing dihydroacetone conversion to dihydroacetone phosphate: ∼5.0 μmol/min/mg. • Specific activity of untagged DhaK catalyzing dihydroacetone conversion to dihydroacetone phosphate: ∼5.1 μmol/min/mg. • Specific activity of immobilized MgsA bearing D18 catalyzing dihydroacetone phosphate conversion to methylglyoxal: ∼14 μmol/min/mg. • Specific activity of immobilized MgsA bearing P18 catalyzing dihydroacetone phosphate conversion to methylglyoxal: ∼13 μmol/min/mg. • Specific activity of untagged Mgs catalyzing dihydroacetone phosphate conversion to methylglyoxal: ∼16 μmol/min/mg. • Specific activity of immobilized GldA bearing D18 catalyzing methylglyoxal conversion to lactaldehyde: ∼0.4 μmol/min/mg. • Specific activity of immobilized GldA bearing P1 catalyzing methylglyoxal conversion to lactaldehyde: ∼0.9 μmol/min/mg. • Specific activity of untagged GldA catalyzing methylglyoxal conversion to lactaldehyde: ∼2.1 μmol/min/mg. • Specific activity of immobilized FucO bearing D18 catalyzing lactaldehyde conversion to 1,2-propanediol: ∼6.0 μmol/min/mg. • Specific activity of immobilized FucO bearing P18 catalyzing lactaldehyde conversion to 1,2-propanediol: ∼2.5 μmol/min/mg. • Specific activity of untagged FucO catalyzing lactaldehyde conversion to 1,2-propanediol: ∼10.0 μmol/min/mg.	• N/A	Lee et al., 2016*
*Salmonella enterica* Pdu bacterial microcompartment SP subunit	β-Galactosidase, β-gal (*E. coli*) (N-terminus) (*Salmonella enterica*) Glycerol dehydrogenase, GldA (*E. coli*) (N-terminus) (*Salmonella enterica*) Esterase, Est5 (soil metagenome) (N-terminus) (*Salmonella enterica*)	• Showed specific activity to their respective substrates but at varying efficiencies. • Catalytic activity of immobilized β-gal catalyzing lactose conversion: 62 ± 7 μmol/h/mg of protein. • Catalytic activity of soluble β-gal catalyzing lactose conversion: 82 ± 7 μmol/h/mg of protein. • Catalytic activity of immobilized β-gal catalyzing o-nitrophenyl-β-galactoside (oNPG) conversion: 4.2 ± 0.17 μmol/h/mg of protein. • Catalytic activity of soluble β-gal catalyzing oNPG conversion: 3.9 ± 0.11 μmol/h/mg of protein. • Catalytic activity of immobilized β-gal catalyzing 4-methylumbelliferyl β-D-galactopyranoside (MUG) conversion: 3.2 × 10^6^ ± 1.8 × 10^5^ relative fluorescence unit (rfu)/min/mg of protein. • Catalytic activity of soluble β-gal catalyzing MUG conversion: 5.0 × 10^6^ ± 1.7 × 10^4^ rfu/min/mg of protein. • Catalytic activity of immobilized GldA catalyzing acetol conversion: 1.1 ± 0.2 μmol/h/mg. • Catalytic activity of soluble GldA catalyzing acetol conversion: 1.4 ± 0.2 μmol/h/mg. • Catalytic activity of immobilized GldA catalyzing methylglyoxal conversion: 1.0 ± 0.1 μmol/h/mg. • Catalytic activity of soluble GldA catalyzing methylglyoxal conversion: 2.1 ± 0.4 μmol/h/mg. • Catalytic activity of immobilized Est5 catalyzing 4-nitrophenyl butyrate (*p*NP-butyrate) conversion: 0.5 ± 0.0 μmol/h/mg. • Catalytic activity of soluble Est5 catalyzing *p*NP-butyrate conversion: 4.3 ± 0.3 μmol/h/mg.	• Enhanced pH stability but not against thermal stress.	Jakobson et al., 2016; Wagner et al., 2017*
*Salmonella enterica* Pdu bacterial microcompartment mutant SP subunit O3-33	Alcohol dehydrogenase D, AdhD (*Pyrococcus furiosus*) (N-terminus) [*E. coli* BL21(DE3)]	• Retained function but at decreased enzyme kinetic activity. • *K*_*m*_ of immobilized AdhD for cofactor NAD^+^: 140 ± 20 μM. • *K*_*m*_ of soluble AdhD for cofactor NAD^+^: 20 ± 7 μM. • *K*_*m*_ of immobilized AdhD for substrate 2,3-butanediol: 140 ± 10 mM. • *K*_*m*_ of soluble AdhD for substrate 2,3-butanediol: 38 ± 8 mM. • Turnover number (*k*_*cat*_) of immobilized AdhD: 0.046 ± 0.002 s^–1^. • *k*_*cat*_ of soluble AdhD: 0.088 ± 0.009 s^–1^. • Apparent *K*_*m*_ of immobilized AdhD for the elctrochemical activity: 28 ± 4 mM. • Apparent *K*_*m*_ of soluble AdhD for the elctrochemical activity: 27 ± 3 mM. • Apparent *k*_*cat*_ of immobilized AdhD for the elctrochemical activity: 0.0084 ± 0.0001 s^–1^. • Apparent *k*_*cat*_ of soluble AdhD for the elctrochemical activity: 0.0086 ± 0.0002 s^–1^.	• Doubled electrochemical operational stability.	Bulutoglu et al., 2019^∧^
*Aquifex aeolicus* Lumazine synthase SP subunit	β-lactamase (*E. coli*) (C-terminus) [*E. coli* BL21(DE3)]	• Enhanced catalytic activity catalyzing nitrocefin hydrolysis at specific configuration.	• N/A	Choi et al., 2018^∧^*
*Thermotoga maritima* Ketohydroxyglutarate aldolase SP subunit	(+)-γ-Lactamase (*Microbacterium hydrocarbonoxydans*) (N-terminus) [*E. coli* BL21(DE3)]	• *K*_*m*_ of immobilized (+)-γ-lactamase catalyzing Vince lactam hydrolysis: 86 ± 2.6 mM. • *K*_*m*_ of soluble (+)-γ-lactamase catalyzing Vince lactam hydrolysis: 120.4 ± 7.2 mM. • *k*_*cat*_ of immobilized (+)-γ-lactamase catalyzing Vince lactam hydrolysis: 12,830 ± 164.5 s^–1^. • *k*_*cat*_ of soluble (+)-γ-lactamase catalyzing Vince lactam hydrolysis: 20088 ± 718 s^–1^.	• Enhanced thermal stability, higher tolerance against organic solvents, proteolysis, and high substrate concentrations.	Li et al., 2018^∧^
*Archaeoglobus fulgidus* Ferritin SP subunit	Kemp eliminase HG3.17 (*Thermoascus aurantiacus*) (N-terminus) [*E. coli* BL21-Gold (DE3)]	• *K*_*m*_ of immobilized HG3.17 catalyzing 5-nitro benzisoxazole degradation: 1400 ± 100 μM. • *K*_*m*_ of soluble HG3.17 catalyzing 5-nitro benzisoxazole degradation: 1700 ± 200μM. • *k*_*cat*_ of immobilized HG3.17 catalyzing 5-nitro benzisoxazole degradation: 150 ± 30 s^–1^. • *k*_*cat*_ of soluble HG3.17 catalyzing 5-nitro benzisoxazole degradation: 170 ± 10 s^–1^. • Specificity constant (*k_*cat*_/K_*m*_*) of immobilized HG3.17 catalyzing 5-nitro benzisoxazole degradation: (11.2 ± 2.5) × 10^4^ M^–1^s^–1^. • *k_*cat*_/K_*m*_* of soluble HG3.17 catalyzing 5-nitro benzisoxazole degradation: (9.9 ± 1.0) × 10^4^ M^–1^s^–1^.	• Showed only partial proteolytic protection after incubation with the blood plasma protease factor Xa. • Immobilized RA95.5-8F showed enhanced thermal stability.	Tetter and Hilvert, 2017^∧^
	Artificial retro-aldolase RA95.5-8F (*Saccharolobus solfataricus* P2) (C-terminus) [*E. coli* BL21-Gold (DE3)]	• *K*_*m*_ of immobilized RA95.5-8F catalyzing (*R*)-4-hydroxy-4-(6-methoxy-2-naphthyl)-2-butanone degradation: 280 ± 30 μM. • *K*_*m*_ of soluble RA95.5-8F catalyzing (*R*)-4-hydroxy-4-(6-methoxy-2-naphthyl)-2-butanone degradation: 300 ± 20 μM. • *k*_*cat*_ of immobilized RA95.5-8F catalyzing (*R*)-4-hydroxy-4-(6-methoxy-2-naphthyl)-2-butanone degradation: 6.2 ± 0.4 s^–1^. • *k*_*cat*_ of soluble RA95.5-8F catalyzing (*R*)-4-hydroxy-4-(6-methoxy-2-naphthyl)-2-butanone degradation: 4.3 ± 0.1 s^–1^. • *k_*cat*_/K_*m*_* of immobilized RA95.5-8F catalyzing (*R*)-4-hydroxy-4-(6-methoxy-2-naphthyl)-2-butanone degradation: (2.2 ± 0.2) × 10^4^ M^–1^s^–1^. • *k_*cat*_/K_*m*_* of soluble RA95.5-8F catalyzing (*R*)-4-hydroxy-4-(6-methoxy-2-naphthyl)-2-butanone degradation: (1.4 ± 0.2) × 10^4^ M^–1^s^–1^.		
	Carbonic anhydrase 2 (*Homo sapiens*) (N-terminus) [*E. coli* BL21-Gold (DE3)]	• *k_*cat*_/K_*m*_* of immobilized carbonic anhydrase 2 catalyzing 4-nitrophenyl acetate degradation: (1.2 ± 0.3) × 10^4^ M^–1^s^–1^. • *k_*cat*_/K_*m*_* of soluble carbonic anhydrase 2 catalyzing 4-nitrophenyl acetate degradation: (1.4 ± 0.4) × 10^3^ M^–1^s^–1^.		
*Myxococcus xanthus* Encapsulin SP subunit	Pyruvate decarboxylase, Aro10p (*Saccharomyces cerevisiae*) (C-terminus) (*Saccharomyces cerevisiae* PK2-1D)	• Decarboxylation activity of immobilized Aro10p catalyzing 4-hydroxyphenylpyruvate conversion to 4-hydroxyphenylacetaldehyde is consistent with its non-immobilized counterpart.	• Enhanced protection against proteolytic degradation.	Lau et al., 2018^∧^*
**Extracellular membrane vesicles (EMVs)**				
Outer membrane vesicles (OMV) via *Pseudomonas syringae* INA5 Ice nucleation protein InaV	Endoglucanase CelA (*Clostridium thermocellum*) (N-terminus) (*E. coli* JC8031) Exoglucanase CelE (*Candida cellulolytica*) (N-terminus) (*E. coli* JC8031) Endoglucanase CelG (*Candida cellulolytica*) (N-terminus) (*E. coli* JC8031)	• Artificial enzyme cascading system comprised of immobilized CelA, CelE, and CelG had enhanced glucose production (∼23-fold higher) compared to its soluble counterpart.	• N/A	Park et al., 2014^∧^*
	Organophosphorus hydrolase, OpdA (*Flavobacterium* sp. strain ATCC 27551) (N-terminus) (*E. coli* JC8031)	• Enhanced paraoxon degradation rate with notable improvement in overall enzyme kinetics upon immobilization. • *K*_*m*_ of immobilized OpdA on OMV catalyzing paraoxon hydrolysis: 42.14 ± 5.22 μM. • *K*_*m*_ of OpdA-OMV immobilized on microcrystalline cellulose catalyzing paraoxon hydrolysis: 51.27 ± 8.14 μM. • *K*_*m*_ of soluble OpdA catalyzing paraoxon hydrolysis: 47.95 ± 9.36 μM. • *k*_*cat*_ of immobilized OpdA on OMV catalyzing paraoxon hydrolysis: 5716 ± 379 s^–1^. • *k*_*cat*_ of OpdA-OMV immobilized on microcrystalline cellulose catalyzing paraoxon hydrolysis: 5579 ± 336 s^–1^. • *k*_*cat*_ of soluble OpdA catalyzing paraoxon hydrolysis: 3513 ± 216 s^–1^. • *k_*cat*_/K_*m*_* of immobilized OpdA on OMV catalyzing paraoxon hydrolysis: 135.64 ± 63.86 μM^–1^s^–1^. • *k_*cat*_/K_*m*_* of OpdA-OMV immobilized on microcrystalline cellulose catalyzing paraoxon hydrolysis: 108.82 ± 18.48 μM^–1^s^–1^. • *k_*cat*_/K_*m*_* of soluble OpdA catalyzing paraoxon hydrolysis: 73.26 ± 19.28 μM^–1^s^–1^.	• Enhanced thermal and pH stability. • Retained at least ∼83% of its initial activity after fifteen reaction cycles. Retained ∼20–30% of its initial activity after 40 days of storage.	Su et al., 2017^∧^
Outer membrane vesicles via *E. coli* outer membrane porin protein OmpA	Phosphotriesterase (*Brevundimonas diminuta*) (C-terminus) [*E. coli* BL21(DE3)]	• Consistent with its soluble counterpart but showed enhanced activity in certain conditions. • *K*_*m*_ of immobilized phosphotriesterase catalyzing paraoxon hydrolysis: 47.3 ± 3.1 μM. • *K*_*m*_ of soluble phosphotriesterase reported in the literature catalyzing paraoxon hydrolysis: 90 μM. • *k*_*cat*_ of immobilized phosphotriesterase catalyzing paraoxon hydrolysis: 2088.7 ± 47.8 s^–1^. • *k*_*cat*_ of soluble phosphotriesterase reported in the literature catalyzing paraoxon hydrolysis: 2400 s^–1^. • *k_*cat*_/K_*m*_* of immobilized phosphotriesterase catalyzing paraoxon hydrolysis: (4.42 ± 0.23) × 10^7^ M^–1^s^–1^. • *k_*cat*_/K_*m*_* of soluble phosphotriesterase reported in the literature catalyzing paraoxon hydrolysis: 2.7 × 10^7^ M^–1^s^–1^.	• Less prone to enzyme inactivation by freezing, lyophilization. • Challenging long-term storage and environment conditions.	Alves et al., 2015, 2016, 2018^∧^
**Magnetosomes**				
Magnetosome membrane protein MamC	Organophosphohydrolase, OpdA (*Flavobacterium* sp. ATCC 27551) (*Magnetospirillum magneticum AMB-1*)	• *K*_*m*_ of immobilized OpdA catalyzing ethyl-paraoxon hydrolysis: 58 ± 2.5 μM. • *K*_*m*_ of soluble OpdA catalyzing ethyl-paraoxon hydrolysis: 43 ± 1.8 μM. • *k*_*cat*_ of immobilized OpdA catalyzing ethyl-paraoxon hydrolysis: 151 ± 6 s^–1^. • *k*_*cat*_ of soluble OpdA catalyzing ethyl-paraoxon hydrolysis: 314 ± 13 s^–1^.	• Stable over six reaction cycles.	Ginet et al., 2011^∧^
	β-glucuronidase (*E. coli*) (C-terminus) (*Magnetospirillum gryphiswaldense*)	• *K*_*m*_ of immobilized β-glucuronidase catalyzing *p*-nitrophenyl-β-D-glucuronide hydrolysis: 0.17 × 10^–3^–0.18 × 10^–3^ M. • *K*_*m*_ of soluble β-glucuronidase catalyzing *p*-nitrophenyl-β-D-glucuronide hydrolysis: 0.28 × 10^–3^ M. • Specific activity of immobilized β-glucuronidase catalyzing *p*-nitrophenyl-β-D-glucuronide hydrolysis: 15.1–16.3 U/mg of enzyme. • Specific activity of soluble β-glucuronidase catalyzing *p*-nitrophenyl-β-D-glucuronide hydrolysis: 12.7 U/mg of enzyme.	• Retained at least ∼75% of its initial activity after 10 reaction cycles.	Mickoleit and Schüler, 2018
Magnetosome membrane protein Mms13	Endoglucanase A (*Clostridium thermocellum*) (C-terminus) (*Magnetospirillum magneticum AMB-1*) β-Glucosidase (*Clostridium thermocellum*) (C-terminus) (*Magnetospirillum magneticum AMB-1*)	• Artificial enzyme cascading system comprised of these two enzymes showed catalytic activity catalyzing the hydrolysis of carboxymethyl cellulose and Avicel. • Co-immobilization of endoglucanase A and β- glucosidase on magnetosomes showed enhanced catalytic activity catalyzing the hydrolysis of carboxymethyl cellulose when compared to the suspension mixture of endoglucanase A immobilized magnetosomes and β-glucosidase immobilized magnetosomes.	• Retained at least ∼70% of its initial activity after five reaction cycles.	Honda et al., 2015a^∧^*

^∧^Specific and/or catalytic activities are not mentioned in the reference. *Kinetic parameters are not mentioned in the reference.

## Comparative Analysis of *In Vivo* Immobilization Strategies

### Advantages and Current Limitations of the Recombinant PHA Particle Technology

Genetic engineering of PAPs represents an interesting approach for enzyme immobilization on PHA particles. Foreign proteins of interest can be translationally fused to the N- or C-terminus, or both termini of PAPs. The broad applicability and versatility of this approach also allows for the attachment of more than one enzyme to the PHA particle surface (Chen S. et al., 2014; Hay et al., 2015; Parlane et al., 2016a). Assembly of immobilized multiprotein complexes enables multi-enzymatic cascade systems with superior catalytic performance as recently reviewed (Hwang and Lee, 2019). Flexible, rigid, and cleavable peptide linkers, such as intein peptide pairs (Du and Rehm, 2017) and LPXTG cleavage sites (sortase A-mediated hydrolysis/ligation) (Du and Rehm, 2018), can be incorporated between the protein functions and PAPs to mediate release of pure target protein (Hay et al., 2014; Rehm et al., 2018). However, underlying molecular mechanisms of PHA particle formation still remain unknown, which intrinsically limits control of their physicochemical properties. For example, a few studies reported that fusing different proteins to PhaC influences the PHA production yield over biomass, particle size distribution, surface charges, and purity of the target protein (Rubio-Reyes et al., 2016; Gonzalez-Miro et al., 2018a, b; Wong and Rehm, 2018). Decorating PHA particles with proteins using PhaC synthase as an anchoring domain can also cause varying distribution and density of respective proteins on the PHA particles (Hay et al., 2015; Wong and Rehm, 2018). Hooks et al. (2013) also pointed out that displaying *N*-acetylneuraminic acid aldolase (NanA) from *E. coli* on PHA particles through N- and C-terminal fusion of PhaC resulted in varying catalytic performance (Hooks et al., 2013). Moreover, similar findings were reported for phasins where fusion of different foreign polypeptides to the BioF tag (PHA-binding domain of PhaF) might have contributed to inconsistency of the physical adsorption function of the BioF-tagged enzyme to the PHA particle surface (Bello-Gil et al., 2018a). A brief comparison of the PHA particle technology with other biological assemblies, detailing their advantages and limitations, is provided in Table 2.

**TABLE 2:** Comparison of PHA particle technology with other biological supramolecular assemblies.

	**Advantages**	**Limitations**
Polyhydroxyalkanoates (PHAs)	• Scalable particle production and able to offer better production yields over biomass • Facile particle functionalization and isolation steps • Structurally very stable • Can be manufactured in a range of recombinant expression systems • Biodegradable • Enhanced shelf-life	• Poor controllability on the physicochemical properties of the particles (e.g., particle size, size distribution, surface charge)—polydisperse and tend to aggregate • Concentration and the function of enzymes localized on the particles, and particle production yield dependent on the folding status of the recombinant fusion proteins
Protein-based particles • Virus-like particles (VLPs) • Enzyme-derived nanoparticles (EZPs)	• Highly programmable physicochemical properties of particles (e.g., particle size, size distribution, surface charge) • Multiple modes of immobilization—tethered within and/or on the surface of particles, and between the CP/SP subunits • Can be manufactured in a range of recombinant expression systems • Biodegradable	• Genetic alteration of CP/SP subunits could trigger structural instability of these scaffolding platforms • Could lead to misfolding of the genetically fused enzymes, especially large domains due to steric hindrance • Labor intensive fabrication processes • Scalability issues • Space available to immobilize functional moieties is limited by the size of the scaffold itself
Extracellular membrane vesicles (EMVs)	• Easy decoration of vesicles • Enzymes of interest can be appended on the surface or within the vesicles • Can be manufactured in a range of recombinant expression systems • Biodegradable	• Poor particle programmability due to the lack of knowledge on the exact assembly mechanism of membrane vesicles • Large-scale consistent production could be difficult • Laborious and expensive isolation procedures
Magnetosomes	• Unique magnetic properties of magnetosomes could be advantageous in some applications (e.g., magnetically driven solid-liquid separation for re-use) • Consistent particle size, particle distribution, and architecture	• Tedious cloning steps and limited design space available for extensive alterations *in vivo* due to potential cell toxicity • Poor controllability in altering the magnetic properties—influenced by the specificity of magnetotatic bacteria • Magnetotatic bacteria are difficult to grow—prolonged production time and low production yields

**FIGURE 3:**
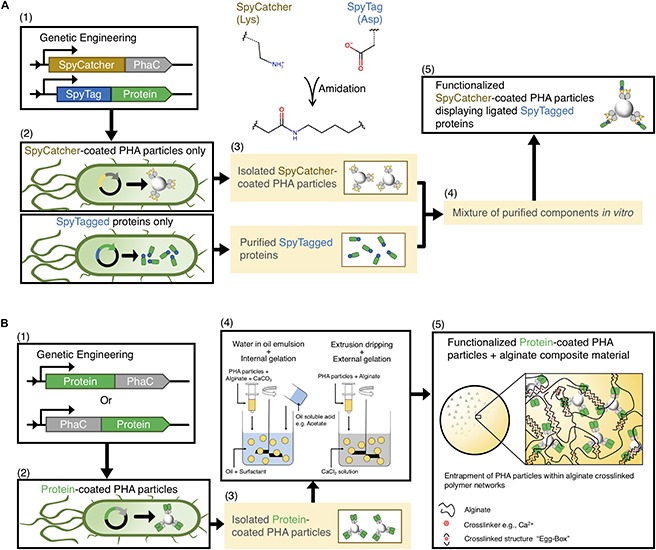
**(A)** Schematic overview of biosynthesis and modular functionalization of SpyCatcher-coated PHA particles. **(B)** Schematic illustration of manufacturing of functionalized PHA particles and subsequent fabrication of alginate–PHA composite materials.

To circumvent these drawbacks regarding the utilization of the PHA particle technology, our group merged the PhaC fusion technology with the SpyTag/SpyCatcher chemistry (Zakeri et al., 2012), which enable better control over production yields and physicochemical properties (Figure 3A) (Wong and Rehm, 2018). We successfully showed that the SpyTagged proteins could ligate to the SpyCatcher-PhaC coated PHA particles *in vitro*, and controlled multifunctionality of PHA particles could be achieved using a sequential immobilization strategy. This approach requires separate and more laborious production of enzymes and scaffold but offers more control over surface coverage and orientation/ratios of the attached proteins. Consistency of the particle size and surface charge of functionalized SpyCatcher-coated PHA particles were observed. Function and conformational stability of the ligated proteins were retained or enhanced (Wong and Rehm, 2018). Recently this approach was expanded by developing streamlined processes exploiting the specificity of the SpyTag/SpyCatcher mediated ligation for efficient and cost-effective modular functionalization (Wong et al., 2020). Overall, PHA particles seem to provide a versatile platform for *in vivo* enzyme immobilization, providing competitive advantages over other biological scaffolds (Table 2). The recent crystal structures, 3D-reconstructed, and homology models of several key PAPs, including *Cupriavidus necator* PhaC and some PhaPs will further inform protein engineering for efficient immobilization of enzymes (Wittenborn et al., 2016; Zhao et al., 2016, 2017; Kim J. et al., 2017; Kim Y. J. et al., 2017).

### Use of Enzyme-Coated PHA Particles in Continuous-Flow Bioprocessing

Immobilized enzymes are widely considered for continuous flow processing toward the synthesis of high-value chemicals (Lee et al., 2015; Contente et al., 2017; Peschke et al., 2017; Döbber et al., 2018; van den Biggelaar et al., 2019). Continuous production of fine chemicals has the potential to accelerate biocatalytic transformations due to enhanced heat and mass transfer between immobilized enzymes and their substrates under flow conditions. The improvement in mass transfer allows the cost-effective miniaturized design of process equipment that ultimately could lead to precise process control and better production yield. Continuous bioprocessing could simplify downstream processing and permit the constant removal of products, such as processes limited by a thermodynamic equilibrium (Tamborini et al., 2018; Thompson et al., 2018). The physical format of the immobilized enzymes needs to be compatible with the continuous-flow process, such as tangential-flow filtration and packed bed/fluidized bed systems (Truppo, 2017).

The lack of uniformity and/or the non-porous properties may restrict the utility of enzyme-coated PHA particles for industrial continuous bioprocesses. Apart from the inherent inconsistency of the PHA particles as outlined above, particulate carriers (<1 μm) are often prone to aggregation under various environmental conditions (e.g., pH, temperature, and ionic strength), which impairs substrate access to the enzymes (Zhang, 2014; Thoniyot et al., 2015) and which could adversely affect their performance in continuous flow processes. The non-porous nature of PHA particles (Hooks et al., 2014) and their tendency to aggregate will cause extensive back-pressure in flow-through applications (Ogura and Rehm, 2019). One innovative solution to overcome these issues is to encapsulate the functionalized PHA particles into a porous hydrogel matrix for efficient integration of enzyme-coated PHA particles into continuous-flow bioprocesses. We recently described an innovative approach that encapsulates functionalized PHA particles within a highly amenable anionic polysaccharide, alginate. The particle–hydrogel composite material was fabricated using the ionotropic gelation method with calcium ion as the cross-linker (Figure 3B) (Ogura and Rehm, 2019). Interestingly, the porosity of the alginate microsphere encapsulating functional protein-coated PHA particles could be controlled by pH during the fabrication process, showing the flexibility of this approach. The various functional protein-coated PHA particles encapsulated within alginate microspheres showed either retained (e.g., organophosphorus hydrolase) or enhanced (e.g., immunoglobulin G-binding ZZ domain) activities in both batch and flow-through mode suggesting suitability for industrial applications (Ogura and Rehm, 2019).

### Potential Industrial Applications of the PHA Particle Technology

There is a widespread agreement that enzyme mediated bioprocesses are environmentally benign as, for example, they reduce consumption of raw materials and energy, while generally able to maintain low levels of waste generation than the traditional non-enzymatic processes (DiCosimo et al., 2013). Implementation of enzymes in large-scale manufacturing could reduce the greenhouse gas emissions when compared to the traditional non-enzymatic processes (Jegannathan and Nielsen, 2013). Therefore, due to the disadvantages in using industrially relevant enzymes in soluble form as mentioned, direct attachment of these enzymes to solid scaffolds, including PHAs, emerged as one of the commercially viable solutions. The advent of PHA particle technology as a generic scaffolding platform for immobilization of enzymes has opened up new routes in developing next-generation catalytic materials for sustainable bioprocessing. We have summarized the recent proof-of-concept demonstrations of the PHA particle technology for industrial applications reported by our group and others (Table 1). Task-specific designer PHA particles can be biosynthesized to serve different industrial applications including the manufacture of commodity chemicals, food products, active pharmaceutical ingredients, and cosmetic chemicals (Abdelraheem et al., 2019). Furthermore, the PHA particle technology can be implemented as a bioremediation tool for the treatment of industrial waste effluents and agricultural pollutants (Sharma et al., 2018).

Since bulk chemicals, such as, e.g., commodity chemicals and food products, are produced at ton scale, high catalytic turnover and ease of reusability of biocatalysts are required for economic feasibility (DiCosimo et al., 2013; Basso and Serban, 2019). Biocatalysts also need to be accessible at low cost and need to be highly stable (Basso and Serban, 2019). On the contrary, different factors need to be considered for production of fine chemicals, such as, e.g., active pharmaceutical ingredients, and cosmetic chemicals, as it is often associated with lower production volumes (e.g., hundreds of kilograms) but higher production yields (Basso and Serban, 2019). More expensive biocatalysts could be considered while the synthesis of these high-value products requires a certain degree of regioselectivity, enantioselectivity, and chemoselectivity (Goldsmith and Tawfik, 2012; Palomo and Guisan, 2012). In addition, the successful implementation of immobilized biocatalysts such as enzyme-coated PHA particles in continuous bioprocessing will be advantageous for production of fine chemicals due to the need for precise process control to achieve the required product quality (Thompson et al., 2018). For bioremediation application, the PHA particle technology offers advantages such as biodegradability of the non-toxic natural PHA scaffold (Ong et al., 2017; Koller, 2018). In recent years, the release of nanoparticles to the environment has sparked some concerns by the research community (Nguyen et al., 2011; Bundschuh et al., 2018).

Given the encouraging proof-of-concept results adapting the PHA particle technology for development of immobilized enzymes for uses in the food industry (Moldes et al., 2004; Rasiah and Rehm, 2009; Ran et al., 2017, 2019), production of commodity chemicals (Jahns and Rehm, 2015; Yang et al., 2015; Seo et al., 2016), production of fine chemicals (Hooks et al., 2013; Chen S. Y. et al., 2014; Tan et al., 2019), and bioremediation (Robins et al., 2013; Hooks and Rehm, 2015; Li et al., 2019), it is anticipated that research prototypes will be developed into industrial products.

## Conclusion and Future Perspectives

Here, we reviewed the advances in the development of several promising biological supramolecular assemblies suitable for *in vivo* enzyme immobilization. We then compared the PHA particle technology with the other scaffolding platforms and discussed innovative strategies to address the challenges associated with developing enzyme-coated PHA particles for industrial applications. Immobilized enzymes exhibit distinct advantages over soluble enzymes, including enhanced stability, improved catalytic performance, reusability, and facilitated product purification. The emergence of biologically inspired particulate carriers has offered promising scaffolding platforms for one-pot *in vivo* enzyme immobilization. Though significant progress has been made to date, numerous challenges, such as high production costs and lack of control over a range of physicochemical properties, need to be tackled to advance these immobilization technologies beyond the proof-of-concept.

As the field of synthetic biology continues to expand rapidly, a more profound understanding of the underlying molecular mechanisms of particle assembly *in vivo* will further inform the rational design of assembled enzyme-carrier systems. The elucidation of these biological processes *in vivo* informs strategies to control several aspects as, for instance, simultaneous PHA particle production and functionalization rational molecular engineering approaches. Such customizable features would allow the creation of, for example, application-specific designer PHA particles for a variety of operating environments. We expect that these remarkable advances can also lay the foundation for the development of monodisperse PHA particles of controllable and reproducible structure and size with programmable surface properties, such as enzyme density/exposure and surface charge. Furthermore, implementing innovative strategies, such as the concept of modularity, fabrication of particle–hydrogel composite materials, and integrated multifunctionality, should increasingly enable implementation in industrial flow-through processes. The development of robust enzyme-carrier systems with porous structures will be critical to ensure implementation for cost-effective continuous biocatalytic conversion and synthesis reactions.

The versatile PHA particle technology offers avenues to immobilize a range of industrially relevant enzymes for development of the next-generation biocatalytic processes. However, the successful “bench-to-factory” translation still requires rigorous optimization and validation to meet industry standards. Additionally, perception barriers as, for instance, the traditional way of thinking and the limited knowledge on sustainable bioprocessing, especially among the manufacturers and regulatory authorities, could hinder the application of these new catalytic materials. Therefore, bridging interdisciplinary boundaries between researchers from the field of molecular biology, chemical engineering, chemistry, and material science should be encouraged. It is critical to integrate diverse methodologies and strategies to further advance *in vivo* enzyme immobilization technologies such as the PHA particle technology.

## Author Contributions

KO wrote section “Enzyme Immobilization for Industrial Applications” of the manuscript. SC wrote section “Utilization of Various Supramolecular Assemblies as Enzyme Immobilization Supports” of the manuscript. JW wrote sections “Biological Supramolecular Assemblies as Biocatalyst Supports” and “Comparative Analysis of *in vivo* Immobilization Strategies” of the manuscript. BR provided critical input in regard to structure, content, and language of the manuscript. All authors provided critical feedback and approved the final version of the manuscript.

## Conflict of Interest

BR is the co-founder and shareholder of PolyBatics Ltd., that commercializes veterinary TB diagnostic products related to the PHA particle technology. The remaining authors declare that the research was conducted in the absence of any commercial or financial relationships that could be construed as a potential conflict of interest.
